# Supplementary material for: Cholesterol metabolism drives regulatory B cell IL-10 through provision of geranylgeranyl pyrophosphate
Source: Nat Commun. 2020 Jul 8;11:3412. doi: 10.1038/s41467-020-17179-4 (PMC7343868; doi:10.1038/s41467-020-17179-4)

Supplementary Information

Cholesterol metabolism drives regulatory B cell IL-10 through provision of geranylgeranyl pyrophosphate

Bibby, J.A. et al.

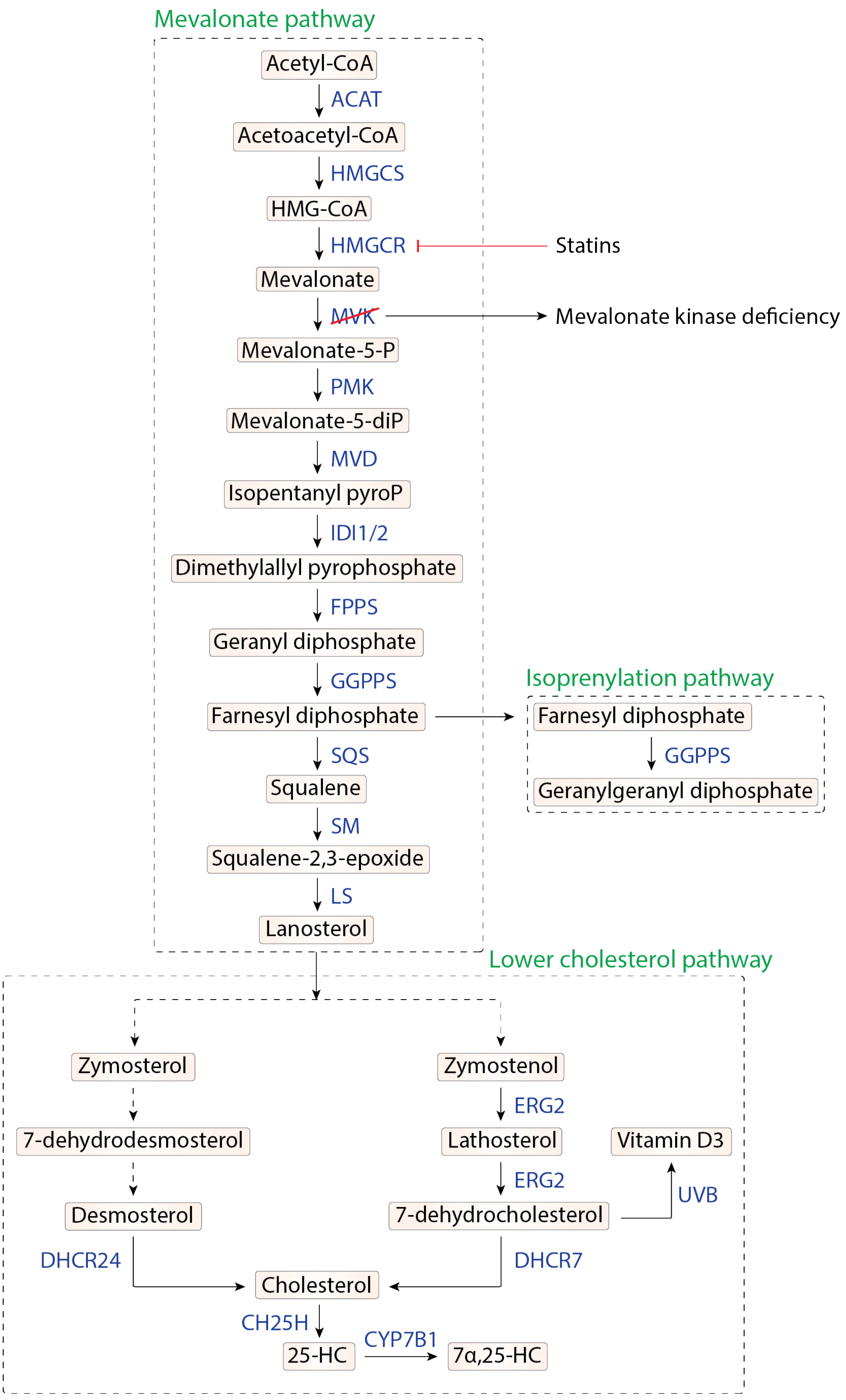

Supplementary Figure 1. Outline of cholesterol metabolism showing key metabolites and enzymes of the pathway. An outline of key metabolites and enzymes in the multi-step conversion of acetyl-CoA to cholesterol and its derivatives, generally termed cholesterol metabolism. Mevalonate kinase deficient patients suffer a mutation in mevalonate kinase (MVK) enzyme, and progress to a severe autoinflammatory syndrome.

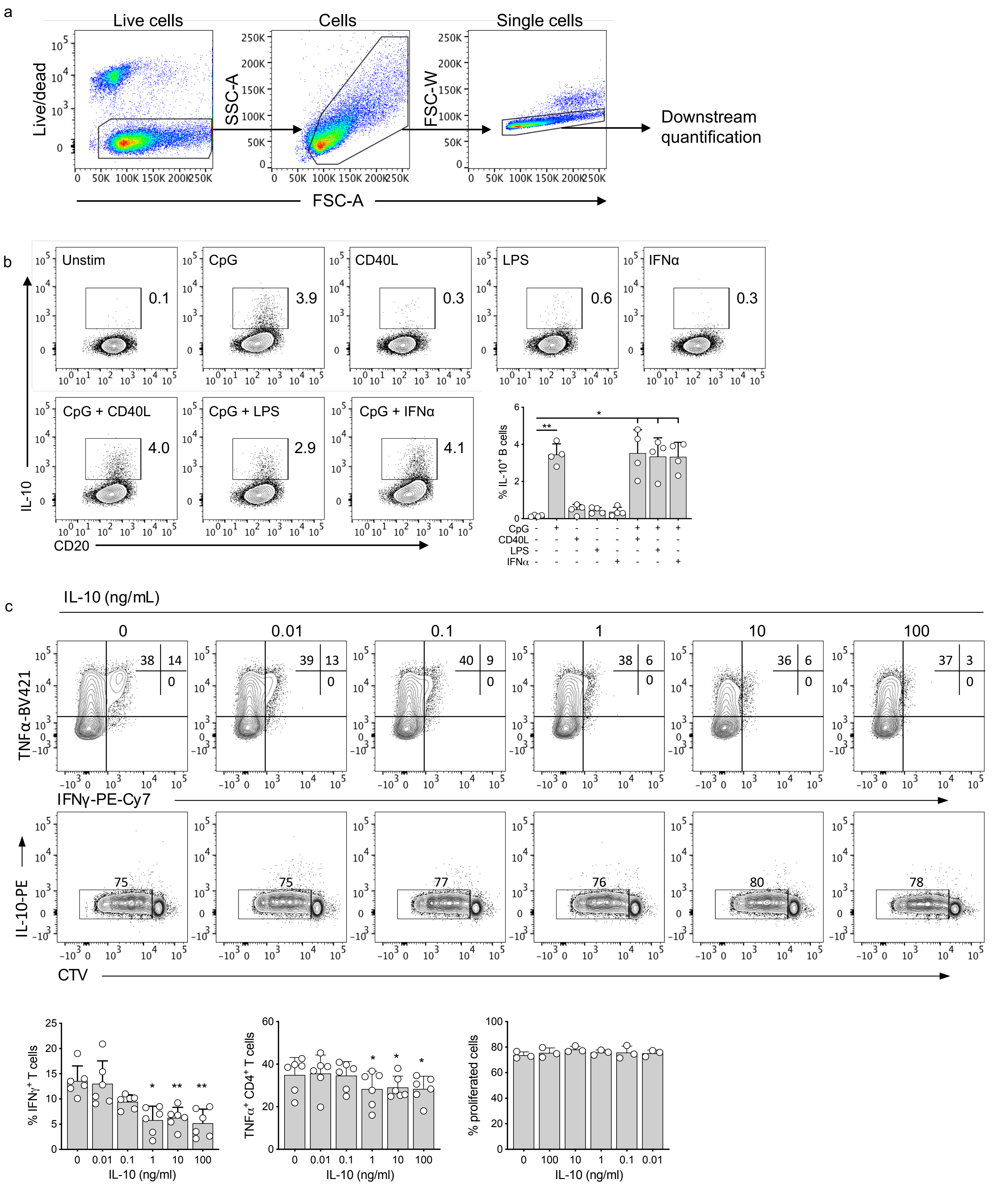

Supplementary Figure 2. IL-10 production by B cells, and the effect of IL-10 on T cell effector function. a. Gating strategy for analysis of human B cells. **b.** IL-10 production by human B cells after stimulation with the indicated ligand for 40 hours. **c.** Representative plots and quantification of IFNγ, TNFα expression, and proliferation in CD4+ T cells after stimulation with anti-CD3/anti-CD28, titration of recombinant human IL-10, and culture for 4 days (n=6 for cytokine measurement, pvals = 0.01, 0.001, 0.001, 0.02, 0.04, 0.02; n=3 for proliferation). Each data point represents individual donors. All data presented are mean ± SD. Statistical testing in all figures was done by a Friedman's test with Dunns's multiple comparisons test. **P*<0.05, ***P*<0.01 and all significant values are shown.

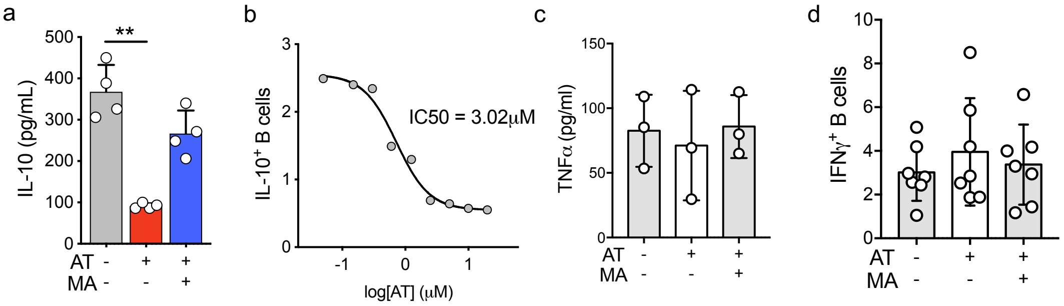

Supplementary Figure 3. Cholesterol metabolism specifically regulates IL-10 expression. a. IL-10 secretion from human B cells after TLR9 stimulation at 40 hours, ± atorvastatin (AT) ± mevalonate (MA) (n=4, pval = 0.005). **b.** IC50 of IL-10 expression in human B cells stimulated through TLR9 in the presence of titrated levels of AT (n=4). **c-d.** Expression of TNFα (ELISA) and IFNγ (flow cytometry) in human B cells stimulated with TLR9 ± AT ± MA (n=3 for TNFα and 7 for IFNγ). Each data point represents individual donors. All data presented are mean ± SD. Statistical testing in all figures was done by a Friedman's test with Dunns's multiple comparisons test. ***P*<0.01 and all significant values are shown.

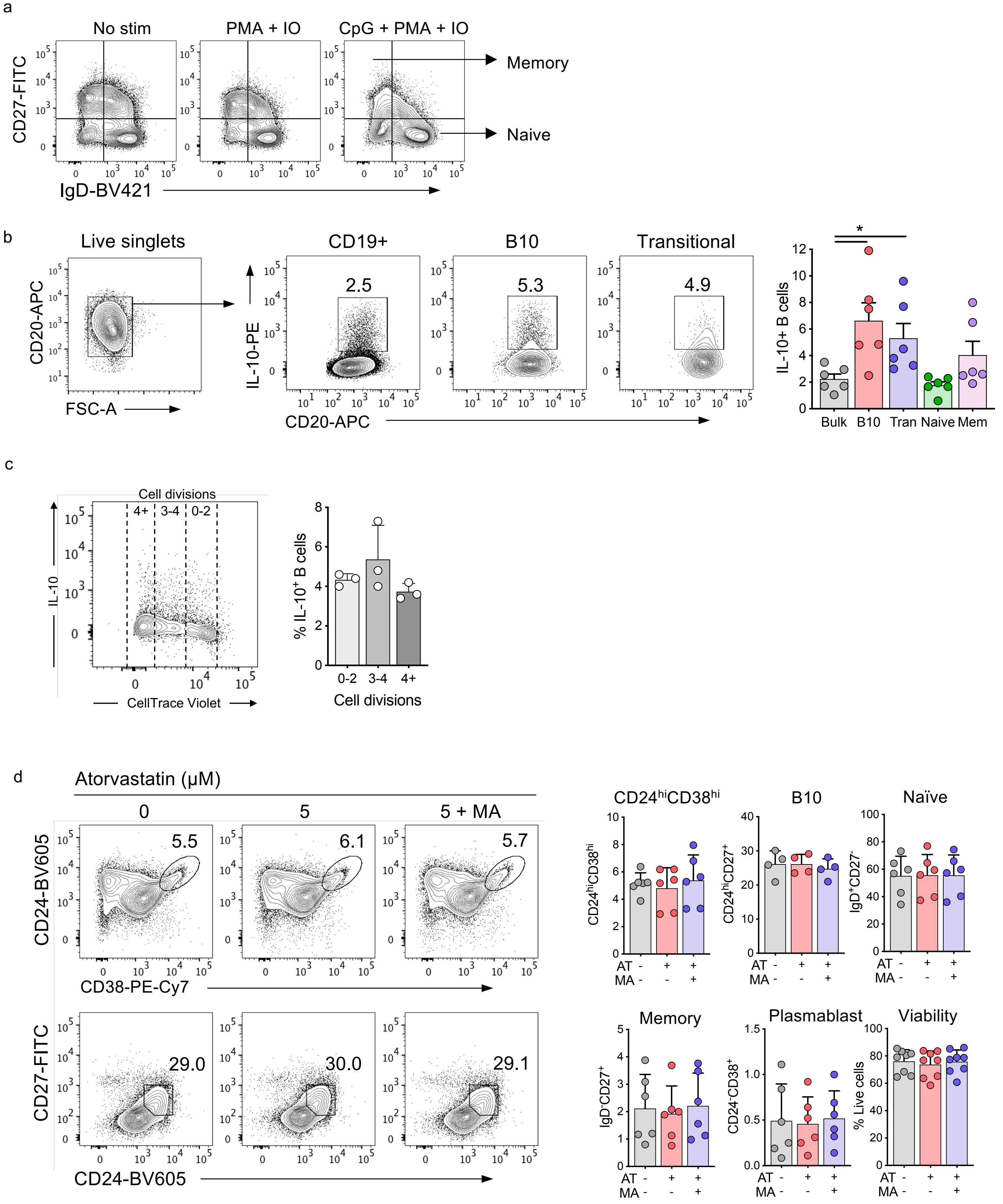
a
No stim
PMA + IO
CpG + PMA + IO
CD27-FITC
IgD-BV421
Memory
Naive
b
Live singlets
CD20-APC
FSC-A
CD19+
B10
Transitional
2.5
5.3
4.9
IL-10-PE
CD20-APC
IL-10+ B cells
*
Bulk
B10
Tran
Naive
Mem
c
Cell divisions
4+
3-4
0-2
IL-10
CellTrace Violet
% IL-10+ B cells
0-2
3-4
4+
Cell divisions
d
Atorvastatin (μM)
0
5
5 + MA
5.5
6.1
5.7
CD24-BV605
CD38-PE-Cy7
29.0
30.0
29.1
CD27-FITC
CD24-BV605
CD24hiCD38hi
B10
Naïve
CD24hiCD27+
IgD+CD27-
AT
MA
Memory
Plasmablast
Viability
IgD-CD27+
CD24-CD38+
% Live cells

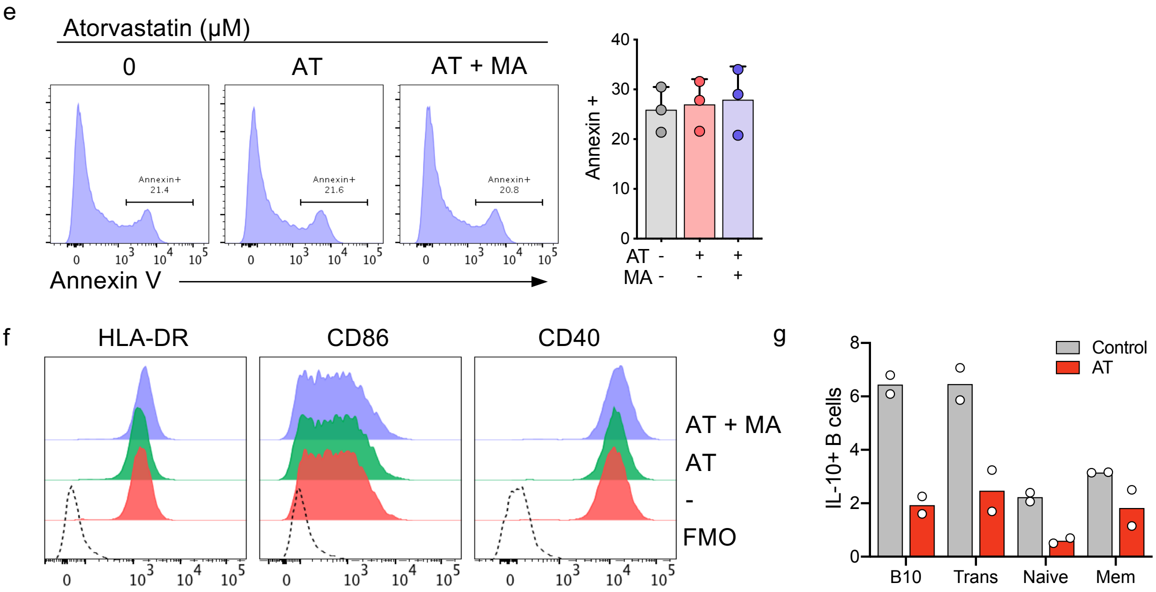

Supplementary Figure 4. IL-10 expression within B cell populations, and the contribution of cholesterol metabolism to B cell phenotype. a. Gating of naïve versus memory B cells after CpG stimulation. CpG stimulation results in the downregulation of CD27, in comparison to unstimulated cells, or phorbol 12-myristate 13-acetate and ionomycin stimulated cells. Memory cells were defined from the upper left quadrant, whereas naïve cells were defined in the lower right. **b**. Percentage of IL-10$^+$ human B cells within each population (bulk (CD19$^+$), B10, CD24^{hi}CD38^{hi}, naïve, and memory) (n=6, pvals = 0.02, 0.05). **c.** IL-10 expression analysed with respect to proliferation, as measured by CellTrace Violet staining prior to stimulation (n=3). **d-e.** Proportional analysis of B cell populations upon stimulation with TLR9 ± atorvastatin (AT) ± mevalonate (MA), and viability measured by live/dead stain (**d,** n=6) or annexin positivity (**e**, n=3). **f.** Representative expression of HLA-DR, CD86, and CD40 on B cells after TLR9 stimulation ± AT ± MA. G**.** IL-10 expression within human B cell populations after stimulation through TLR9 ± AT. **g.** IL-10 expression in B cell populations following CpG stimulation in the presence or absence of AT. Each data point represents individual donors. All data presented are mean ± SD. Statistical testing in all figures was done by a Friedman's test with Dunns's multiple comparisons test. **P*<0.05 and all significant values are shown.

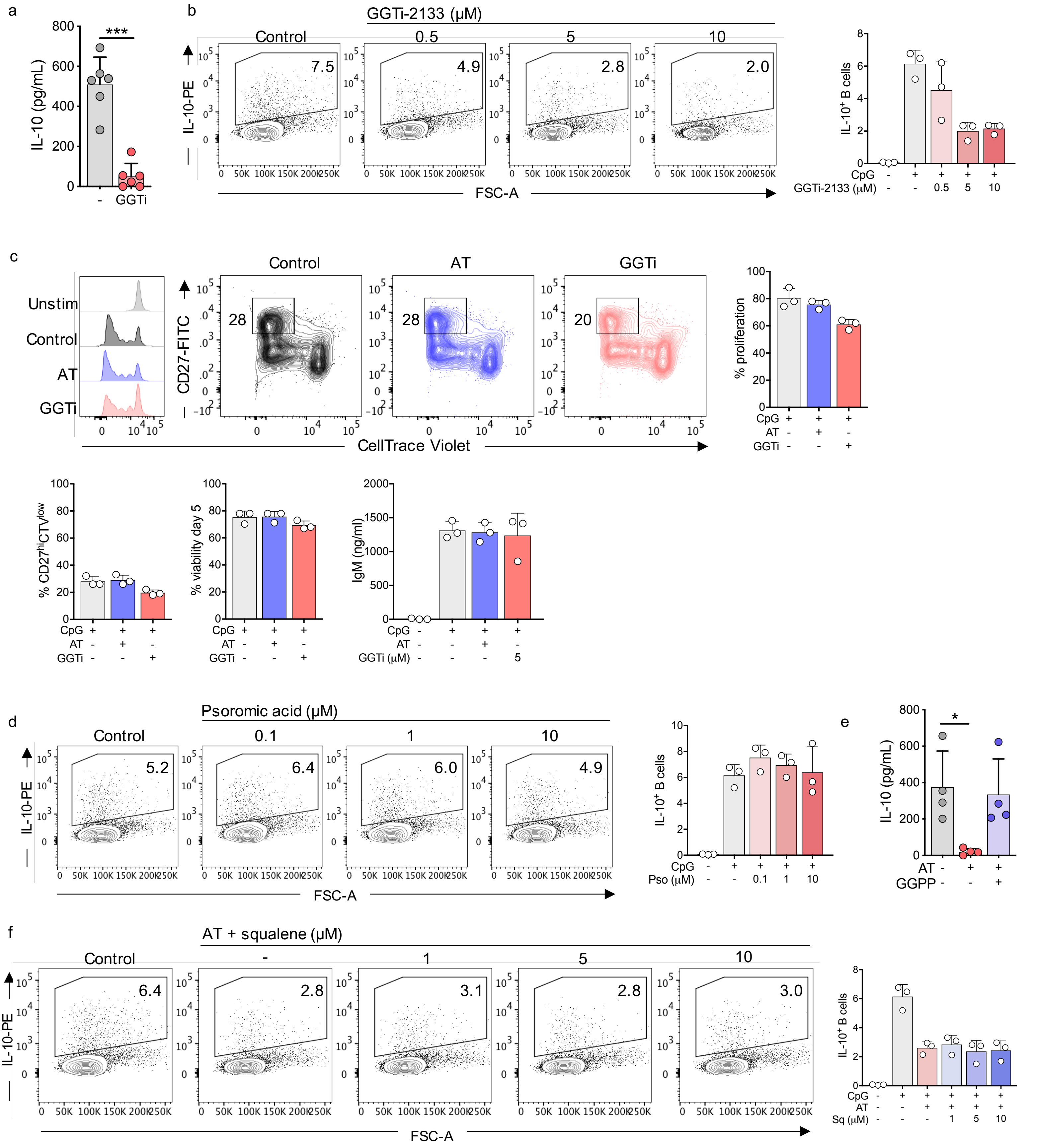

Supplementary Figure 5. Cholesterol metabolism regulates IL-10 via GGTase and GGPP. a. IL-10 secretion in TLR9 stimulated human B cells in the presence of geranylgeranyl transferase inhibition (GGTi), measured by ELISA (n=6, pval = 0.001). **b.** IL-10 expression in human B cells after stimulation through TLR9 ± GGTi-2133 (n=3). **c.** Proliferation, differentiation, viability (all at day 5), and IgM production (at day 7) by human B cells after stimulation through TLR9 ± AT ± GGTi-2133 (n=3). **d-f.** IL-10 production in TLR9 stimulated human B cells ± psoromic acid (**d**, n=3), ± atorvastatin (AT) ± geranylgeranyl pyrophosphate (GGPP) (**e,** n=4, pva = 0.03), or ± AT ± Squalene (**f,** n=3). Each data point represents individual donors. All data presented are mean ± SD. Statistical testing in (a) was done by a paired t test, or in (e) by a Friedman's test with Dunns's multiple comparisons test. **P*<0.05, ****P*<0.001 and all significant values are shown.

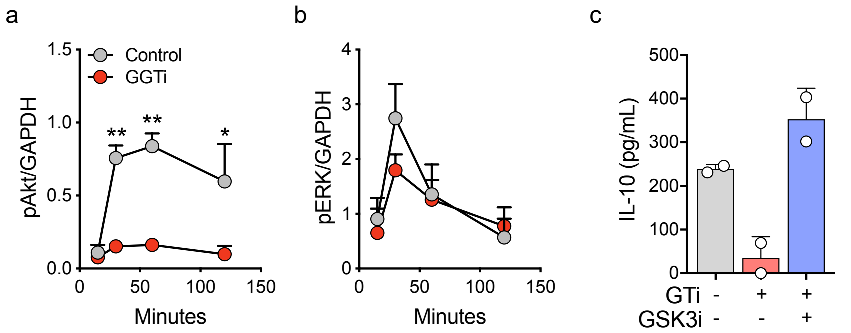

Supplementary Figure 6. GGTase and GGPP regulate IL-10 induction downstream of TLR9 via PI3K, AKT, and ERK. a-b. Quantification of AKT **(a)** and ERK **(b)** phosphorylation over time in human B cells after stimulation through TLR9 ± geranylgeranyl transferase inhibitor (GGTi) (both n=3, pvals = 0.004, 0.001, 0.01). **c.** IL-10 secretion from human B cells after stimulation through TLR9 ± GGTi ± GSK3i (n=2). All data presented are mean ± SD. Statistical testing in all figures was done by a two-way ANOVA with Sidak's multiple comparisons test. *P<0.05, **P<0.01 and all significant values are shown.

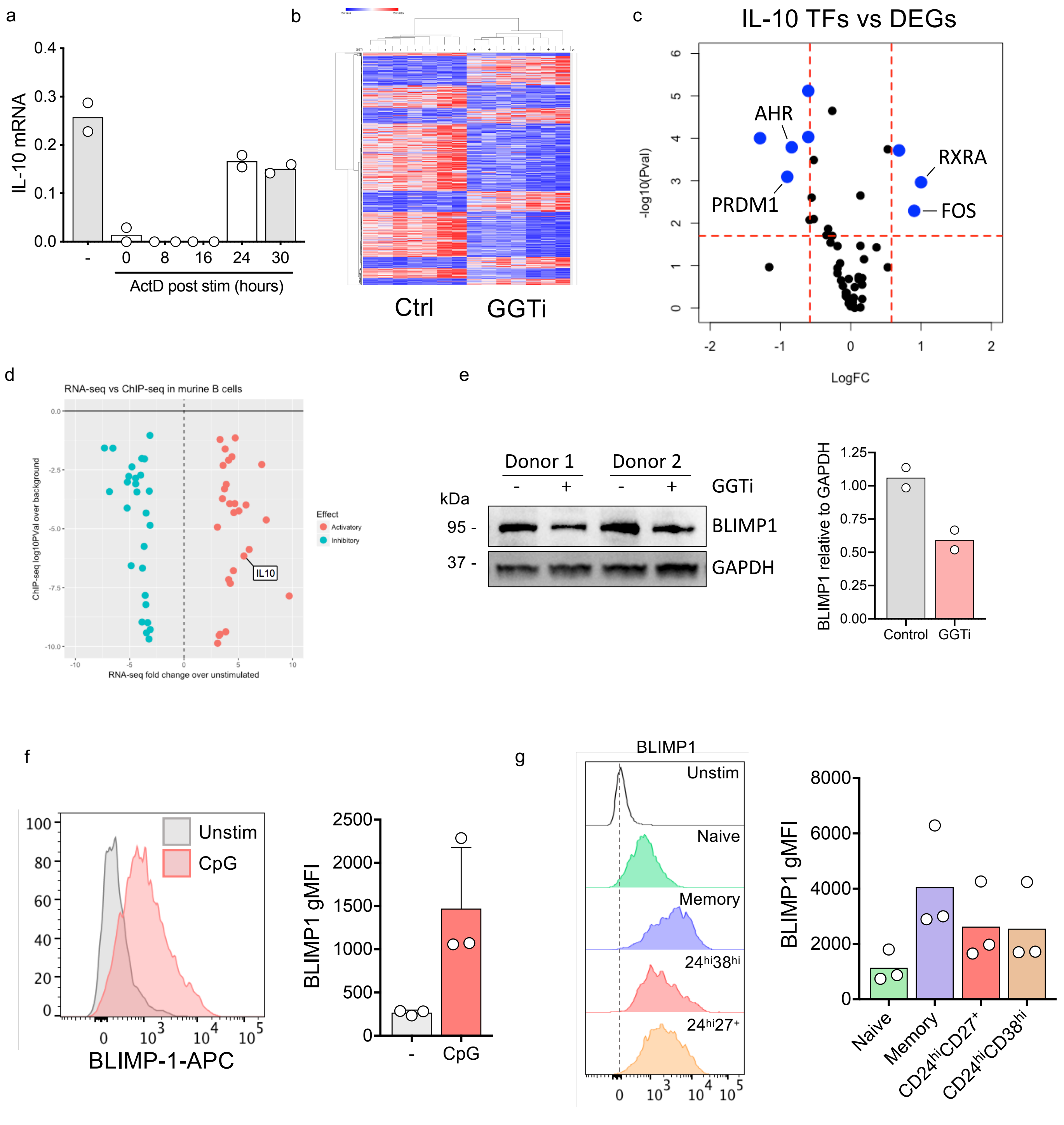

Supplementary Figure 7. A transcriptional event mediated by GGTase after TLR9 stimulation is required for the expression of a transcription factor necessary for IL-10. a. qRT-PCR analysis of IL-10 expression in B cells following TLR9 stimulation ± actinomysin D (ActD) added at the indicated time post-stimulation, with all cells acquired at 40 hours (n=2). **b.** A heatmap representing the global profile of differentially expressed genes (FDR<0.05, Fold change >1.5) in human B cells following stimulation through TLR9 ± geranylgeranyl transferase inhibition (GGTi). **c.** All previously experimentally validated IL-10 transcription factors, and their FDR and fold change in our data set. Red dashed lines represent FDR=0.05 and fold change=1.5. **d.** ChIP-seq (y-axis) vs RNA-seq (x-axis) data from GSE71698, showing enriched targets of BLIMP1, and the correlative change in gene expression. **e.** BLIMP1 protein levels after B cells were stimulated with CpG in the presence or absence of GGTi (n=2). **(f-g)** Expression of BLIMP1 in human B cells at 40 hours after stimulation through TLR9, either in bulk B cells (**f,** n=3), or in different B cell phenotypes (**g,** n=3). Each data point represents individual donors. All data presented are mean ± SD. FDR values were calculated in EdgeR using the default Benjamini-Hochberg correction

a

Patient	Status	Mutation	Sex	Age
MKD1	MKD	V377I/I268T	M	26
HD1	Healthy	NA	M	27
MKD2	MKD	V377I/H380R	M	29
HD2	Healthy	NA	M	32
MKD3	MKD	V377I/G/G202R	M	26
HD3	Healthy	NA	M	22
MKD4	MKD	V377I//H380R	M	27
HD4	Healthy	NA	M	27
MKD5	MKD	V377I/R215X	M	26
HD5	Healthy	NA	M	27
MKD6	MKD	V377I/I268T	F	7
HD6	Healthy	NA	M	40

b

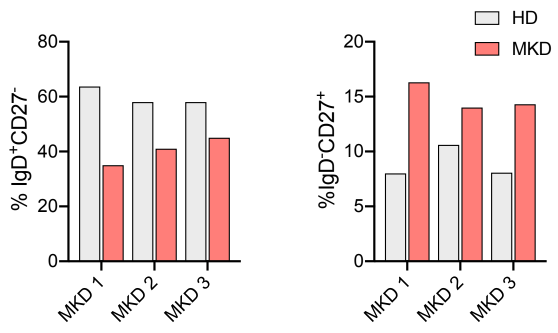

c

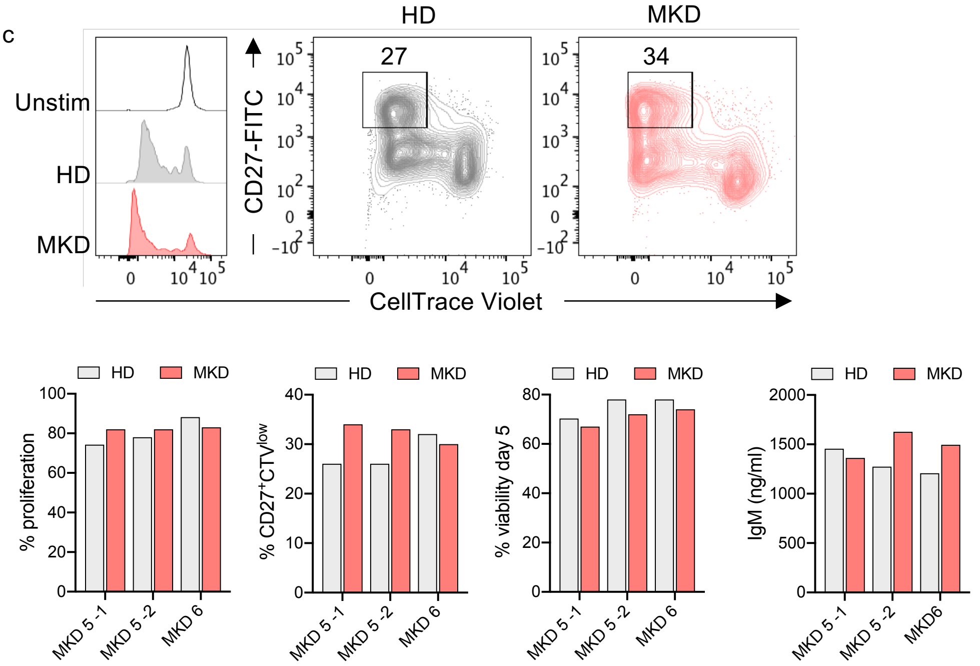

d

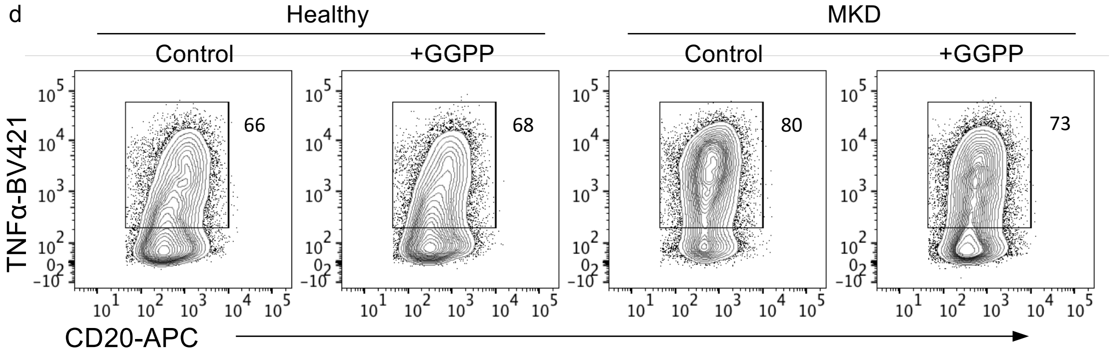

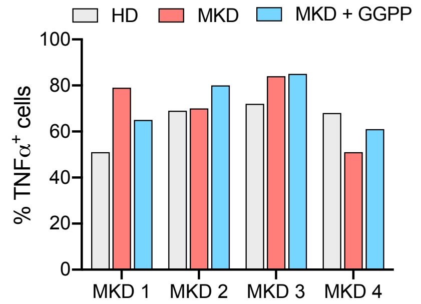

e

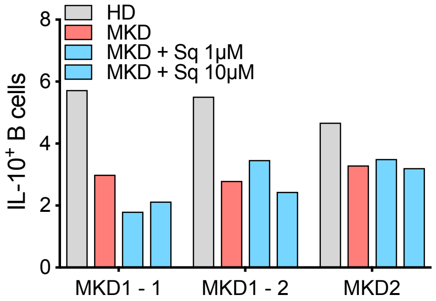

f

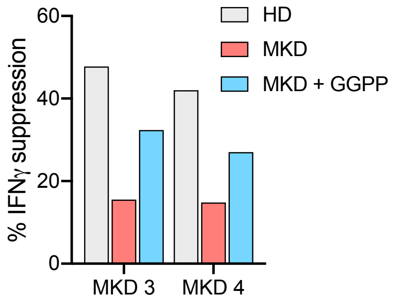

g

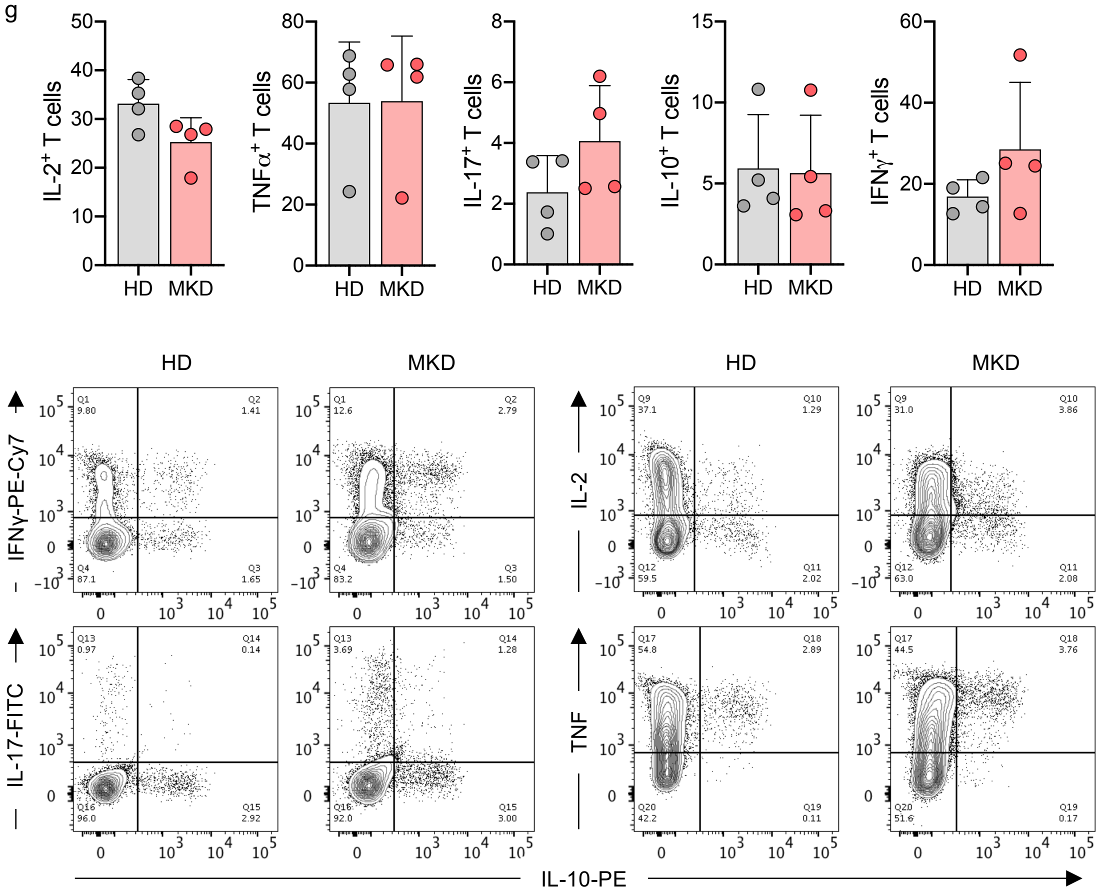

Supplementary Figure 8. MKD patients show poor regulatory B cell responses, but normal T cell responses. a. Clinical information for healthy donors (HD) and mevalonate kinase deficient (MKD) patients used in this study. **b.** Naive versus memory B cell populations in MKD patients relative to healthy donors, as a percentage of total $CD19^+$ B cells. **c.** Proliferation, differentiation, viability (at day 5), and IgM production (at day 7) by HD or MKD patient B cells after stimulation through TLR9. Technical repeats shown for MKD 5 as suffixed with -1 and -2. **d.** TNFα expression in human B cells from HD or MKD patients stimulated through TLR9 ± geranylgeranyl pyrophosphate (GGPP). **e.** IL-10 expression in B cells from MKD patients and HD after stimulation through TLR9 ± squalene (sq). Technical repeats shown for MKD 5 as suffixed with -1 and -2. **f.** IFNγ suppression in human $CD4^+$ T cells of healthy controls and MKD patients after co-culture with autologous TLR9 activated B cells ± GGPP treated B cells prior to co-culture. **g**. Cytokine production in $CD4^+$ T cells from MKD patients and healthy controls (n=4). Each data point represents individual donors. All data presented are mean ± SD. All significant values are shown.

Figure 3d

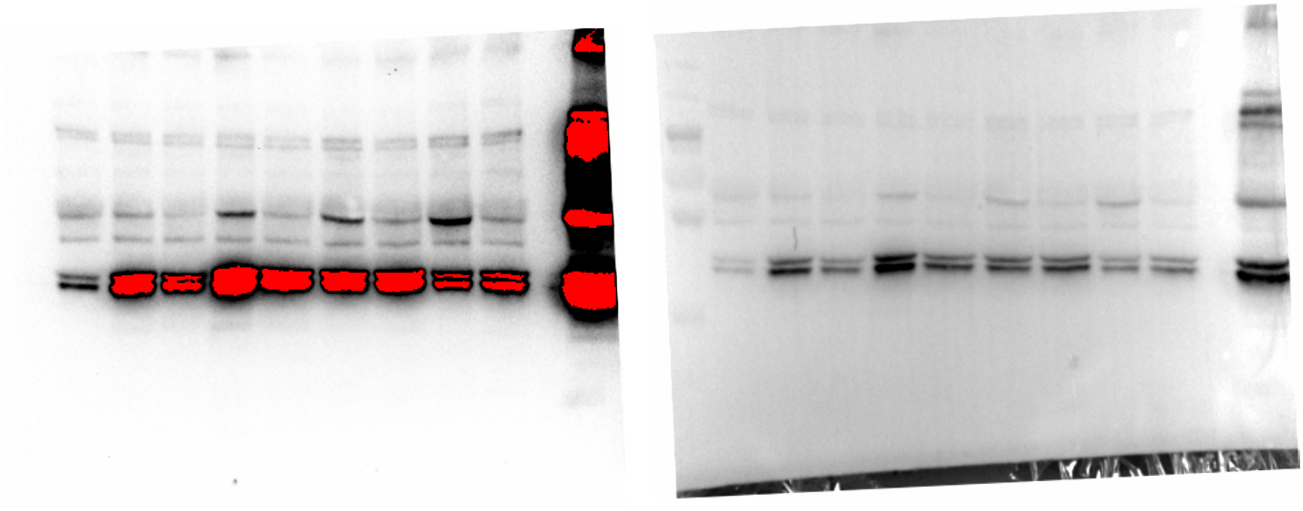

Figure 5f and g

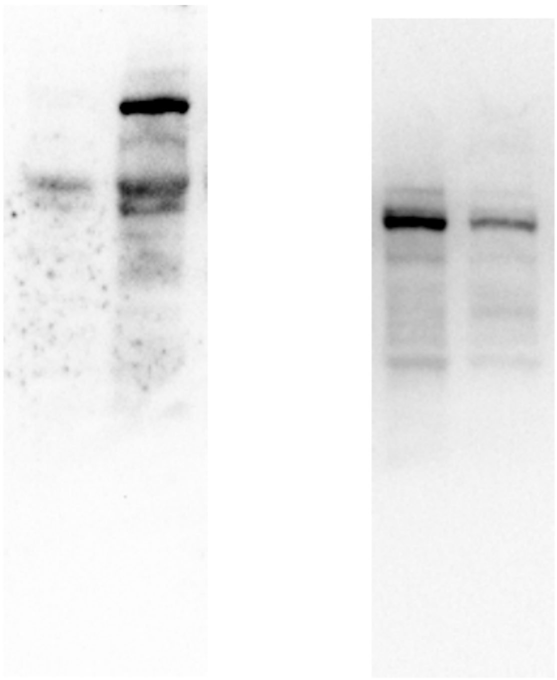

Sup Fig 7e

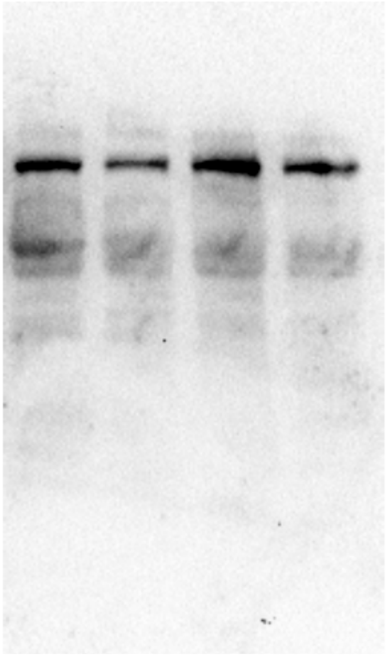

Supplement: Supplementary file 1 — Supplementary Information [file 41467_2020_17179_MOESM1_ESM.pdf]
