# Supplementary material for: Cholesterol metabolism drives regulatory B cell IL-10 through provision of geranylgeranyl pyrophosphate
Source: Nat Commun. 2020 Jul 8;11:3412. doi: 10.1038/s41467-020-17179-4 (PMC7343868; doi:10.1038/s41467-020-17179-4)
Supplement: Supplementary file 3 — Reporting Summary [file 41467_2020_17179_MOESM3_ESM.pdf]

nature research

Corresponding author(s): Jack Bibby

Last updated by author(s): May 10, 2020

Reporting Summary

Nature Research wishes to improve the reproducibility of the work that we publish. This form provides structure for consistency and transparency in reporting. For further information on Nature Research policies, see our Editorial Policies and the Editorial Policy Checklist.

Statistics

For all statistical analyses, confirm that the following items are present in the figure legend, table legend, main text, or Methods section.

n/a	Confirmed	
☐	☒	The exact sample size (n) for each experimental group/condition, given as a discrete number and unit of measurement
☐	☒	A statement on whether measurements were taken from distinct samples or whether the same sample was measured repeatedly
☐	☒	The statistical test(s) used AND whether they are one- or two-sided<br>*Only common tests should be described solely by name; describe more complex techniques in the Methods section.*
☒	☐	A description of all covariates tested
☐	☒	A description of any assumptions or corrections, such as tests of normality and adjustment for multiple comparisons
☒	☐	A full description of the statistical parameters including central tendency (e.g. means) or other basic estimates (e.g. regression coefficient) AND variation (e.g. standard deviation) or associated estimates of uncertainty (e.g. confidence intervals)
☐	☒	For null hypothesis testing, the test statistic (e.g. F, t, r) with confidence intervals, effect sizes, degrees of freedom and P value noted<br>*Give P values as exact values whenever suitable.*
☒	☐	For Bayesian analysis, information on the choice of priors and Markov chain Monte Carlo settings
☒	☐	For hierarchical and complex designs, identification of the appropriate level for tests and full reporting of outcomes
☒	☐	Estimates of effect sizes (e.g. Cohen's d, Pearson's r), indicating how they were calculated

Our web collection on statistics for biologists contains articles on many of the points above.

Software and code

Policy information about availability of computer code

Data collection	No software used
Data analysis	GSEA (v4.0.2 for Mac), GraphPad Prism (v7.0), R (v3.6.3), RStudio (v1.0.153), Morpheus (https://software.broadinstitute.org/morpheus)

For manuscripts utilizing custom algorithms or software that are central to the research but not yet described in published literature, software must be made available to editors and reviewers. We strongly encourage code deposition in a community repository (e.g. GitHub). See the Nature Research guidelines for submitting code & software for further information.

Data

Policy information about availability of data

All manuscripts must include a data availability statement. This statement should provide the following information, where applicable:
- Accession codes, unique identifiers, or web links for publicly available datasets
- A list of figures that have associated raw data
- A description of any restrictions on data availability

RNA-sequencing generated in this study can be found at: GSE150245. Publicly available datasets used in the analysis can be found at GSE43553 (used in GSEA analysis), and GSE71698 (used for ChIP and RNA-seq analysis)

Field-specific reporting

Life sciences study design

All studies must disclose on these points even when the disclosure is negative.

Sample size	Sample sizes were restricted to the number of available patient samples and therefore could not be calculated. Total patient and healthy donor control samples used in the study = 6. Each experiment was repeated at least three times where possible to generate sufficient power for statistical analysis.
Data exclusions	No data were excluded from the analyses
Replication	Biological replicates are included in all experiments, and success of replication was tested using the appropriate statistical analysis indicated in the figure legend
Randomization	Randomization was not used as the data were conducted in vitro with repeated human samples
Blinding	No blinding was conducted during data acquisition or analysis as the same person performed and analyzed experiments

Reporting for specific materials, systems and methods

We require information from authors about some types of materials, experimental systems and methods used in many studies. Here, indicate whether each material, system or method listed is relevant to your study. If you are not sure if a list item applies to your research, read the appropriate section before selecting a response.

Materials & experimental systems

n/a	Involved in the study	
	☒	Antibodies
☒		Eukaryotic cell lines
☒		Palaeontology and archaeology
	☒	Animals and other organisms
	☒	Human research participants
☒		Clinical data
☒		Dual use research of concern

Methods

n/a	Involved in the study	
☒		ChIP-seq
	☒	Flow cytometry
☒		MRI-based neuroimaging

Antibodies

Antibodies used	IL-10-PE (JES3-19F1), TNFa-BV421 (Mab11), IFNy-PE/Cy7 (4S.B3), CD20-AF647 (2H7), CD24-BV605 (ML5), CD27-FITC (M-T271), CD38-PE/Cy7 (HB-7), IgD-BV421 (IA6-2), CD4-FITC (A161A1), HLA-DR-PerCP/Cy5.5 (L243), CD86-BV421 (IT2.2), CD40-PE (5C3): all Biolegend, pGSK3ser9-PE (REA436, Miltenyi), and BLIMP1-APC and BLIMP1 (646702, R&D Systems), pERK (#9101), pAktser473 (D9E) both Cell Signaling Technology.
Validation	Antibodies used were titrated prior to use, with their dilution indicated in the methods section.<br>References of antibodies used in this study are as follows:<br>- IL-10-PE (JES3-19F1) Shey M, et al. 2014. J Immunol. 192:4833<br>- TNFa-BV421 (Mab11) Montel-Hagen A et al. 2019. Cell stem cell. 24(3):376-389<br>- CD20-AF647 (2H7) Ven A, et al. 2012. J Allergy Clin Immunol. 129:755<br>- CD24-BV605 (ML5) Kristiansen G, et al. 2003. Clin. Cancer Res. 9:4906<br>- CD27-FITC (M-T271) Alivernini S, et al. 2016. Nat Commun. 7: 12970<br>- IgD-BV421 (IA6-2) Tardif V, et al. 2019. Nat Commun. 10:823<br>- CD4-FITC (A161A1) Angela M, et al. 2016. Nat Commun. 7:13683<br>- HLA-DR-PerCP/Cy5.5 (L243) Price A, et al. 2016. Proc Natl Acad Sci U S A. 113: 10678 - 1068<br>- CD86-BV421 (IT2.2) Zhao Y, et al. 2019. Immunity. 51:1059<br>- CD40-PE (5C3) Škrnjug I, et al. 2014. PLoS One. 9:110150<br>- pGSK3ser9-PE (REA436, Miltenyi) Giovannini, C. et al. (2013) Oncotarget 4(10): 1618-1631<br>- BLIMP1-APC and unconjugated (646702, R&D Systems) Manuf website: "Detects human BLIMP1/PRDM1 in Western blots" including validation images<br>- pERK (#9101) Franke, T.F. et al. (1997) Cell 88, 435-7<br>- pAktser473 (D9E) Marais, R. et al. (1993) Cell 73, 381-93.

Animals and other organisms

Policy information about studies involving animals; ARRIVE guidelines recommended for reporting animal research

Laboratory animals	Total splenocytes were isolated from 8-15-week-old male and female mice, as described in methods section. Both the D910A and E1020K mice were back-crossed to the C57BL/6 background.

Wild animals	No wild animals were used
Field-collected samples	No field collected samples were used
Ethics oversight	Animal experiments were performed according to the Animals (Scientific Procedures) Act 1986, license PPL 70/7661 and approved by the Babraham Institute Animal Welfare and Ethics Review Body.

Note that full information on the approval of the study protocol must also be provided in the manuscript.

Human research participants

Policy information about studies involving human research participants

Population characteristics	Data concerning patient information can be found in supplementary Figure 8
Recruitment	All patients were recruited during routine clinic visits. Due to the unpredictable nature of the disease and rarity of the patients, we were not able to control the exact timing of sample collection.
Ethics oversight	Written informed consent was received from all healthy donors and patients used in this study, approved by the Bromley Research Ethics Committee (REC06/Q0705/20) and by the NIDDK/NIAMS Institutional Review Board

Note that full information on the approval of the study protocol must also be provided in the manuscript.

Flow Cytometry

Plots

Confirm that:

- [x] The axis labels state the marker and fluorochrome used (e.g. CD4-FITC).
- [x] The axis scales are clearly visible. Include numbers along axes only for bottom left plot of group (a 'group' is an analysis of identical markers).
- [x] All plots are contour plots with outliers or pseudocolor plots.
- [x] A numerical value for number of cells or percentage (with statistics) is provided.

Methodology

Sample preparation	For intracellular cytokine detection, cells were restimulated in the final 3 (human) or 5 (mice) hours of culture with phorbol 12-myristate 13-acetate (50ng/mL, Sigma) and ionomycin (500ng/mL, Sigma) in the presence of Brefeldin A, and GolgiStop (both 1ul/ml, both BD Biosciences). For surface staining, cells were harvested and incubated with Fixable Viability Dye eFluor780 (eBiosciences) for 15 minutes in phosphate buffered saline (PBS), followed by the appropriate volume of antibody diluted in 0.5% bovine serum albumin (BSA) in PBS for 20 minutes, all at 4C. Cells were then washed and fixed in 3% paraformaldehyde (Electron Microscopy Sciences) for 15 minutes at room temperature. For intracellular cytokine staining, cells were incubated with the appropriate volume of antibody, diluted in 0.1% saponin in 0.5% BSA in PBS for 45 minutes at room temperature or 4C overnight. For staining of transcription factors, or phospho-flow, FoxP3/Transcription factor buffer set was used, as per manufacturer's instructions (eBiosciences). Staining using a secondary antibody against the primary was undertaken either at room temperature for 1 hour, or 4C overnight.
Instrument	Cells were acquired using a BD LSRFortessa or FACSCanto II (BD Biosciences)
Software	FlowJo V.10.1 software (Tree Star Inc.)
Cell population abundance	No cells underwent sorting
Gating strategy	FCS-A v SSC-A > FSC-A v FSC-W > Live/Dead negative > downstream quantification

- [x] Tick this box to confirm that a figure exemplifying the gating strategy is provided in the Supplementary Information.
